# High-quality permanent draft genome sequence of the extremely osmotolerant diphenol degrading bacterium *Halotalea alkalilenta* AW-7^T^, and emended description of the genus *Halotalea*

**DOI:** 10.1186/s40793-015-0052-7

**Published:** 2015-08-13

**Authors:** Spyridon Ntougias, Alla Lapidus, Alex Copeland, T. B. K. Reddy, Amrita Pati, Natalia N. Ivanova, Victor M. Markowitz, Hans-Peter Klenk, Tanja Woyke, Constantinos Fasseas, Nikos C. Kyrpides, Georgios I. Zervakis

**Affiliations:** Laboratory of Wastewater Management and Treatment Technologies, Department of Environmental Engineering, Democritus University of Thrace, Xanthi, Greece; Theodosius Dobzhansky Center for Genome Bioinformatics, St. Petersburg State University, St. Petersburg, Russia; Algorithmic Biology Lab, St. Petersburg Academic University, St. Petersburg, Russia; Department of Energy Joint Genome Institute, Genome Biology Program, Walnut Creek, CA USA; Biological Data Management and Technology Center, Lawrence Berkeley National Laboratory, Berkeley, CA USA; Leibniz Institute DSMZ – German Collection of Microorganisms and Cell Cultures, Braunschweig, Germany; Electron Microscopy Laboratory, Agricultural University of Athens, Athens, Greece; Department of Biological Sciences, Faculty of Science, King Abdulaziz University, Jeddah, Saudi Arabia; Laboratory of General and Agricultural Microbiology, Agricultural University of Athens, Athens, Greece

**Keywords:** Alkaline two-phase olive mill waste, *Halomonadaceae*, Protocatechuate ortho-cleavage, Catechol to β-ketoadipate degradation pathway, Cyanate and acrylonitrile detoxification, GEBA-KMG

## Abstract

Members of the genus *Halotalea* (family *Halomonadaceae*) are of high significance since they can tolerate the greatest glucose and maltose concentrations ever reported for known bacteria and are involved in the degradation of industrial effluents. Here, the characteristics and the permanent-draft genome sequence and annotation of *Halotalea alkalilenta* AW-7^T^ are described. The microorganism was sequenced as a part of the Genomic Encyclopedia of Type Strains, Phase I: the one thousand microbial genomes (KMG) project at the DOE Joint Genome Institute, and it is the only strain within the genus *Halotalea* having its genome sequenced. The genome is 4,467,826 bp long and consists of 40 scaffolds with 64.62 % average GC content. A total of 4,104 genes were predicted, comprising of 4,028 protein-coding and 76 RNA genes. Most protein-coding genes (87.79 %) were assigned to a putative function. *Halotalea alkalilenta* AW-7^T^ encodes the catechol and protocatechuate degradation to β-ketoadipate via the β-ketoadipate and protocatechuate ortho-cleavage degradation pathway, and it possesses the genetic ability to detoxify fluoroacetate, cyanate and acrylonitrile. An emended description of the genus *Halotalea* Ntougias et al. 2007 is also provided in order to describe the delayed fermentation ability of the type strain.

## Introduction

The genus *Halotalea* includes a single species*, i.e.*, *H. alkalilenta**,* which is a motile, rod-shaped, alkalitolerant and halotolerant Gram-negative staining heterotrophic bacterium [1]. Strain AW-7^T^ (=DSM 17697^T^ =CECT 7134^T^ =CIP 109710^T^) is the type species of the genus *Halotalea* and of the type strain of the species *H. alkalilenta* [1]. The strain was isolated from alkaline olive mill waste, which was generated by a two-phase centrifugal olive oil extraction system located in the Toplou Monastery area, Sitia, Crete [1]. The Neo-Latin genus name derived from the Greek and the Latin nouns *halos* and *talea*, meaning salt-living and rod-shaped cells, respectively. The Neo-Latin species epithet *halotalea* composed of the Arabic term *al qaliy* and the Latin epithet *lentus* (*a*), meaning alkali and slow respectively which refer to slowly-growing cells under alkaline conditions (alkalitolerant) [1].

*Halotalea alkalilenta* belongs to the family *Halomonadaceae* [1–4], which has accommodated in chronological order the genera *Halomonas* [5], *Chromohalobacter* [6], *Zymobacter* [7], *Carnimonas* [8], *Cobieta* [9], *Halotalea* [1], *Modicisalibacter* [4], *Salinicola* [10], *Kushneria* [11], *Aidingimonas* [12] and *Larsenimonas* [13–15]. By employing multilocus sequence analysis, de la Haba et al. [16] found that all genera of the family *Halomonadaceae*, apart from *Halomonas* and *Modicisalibacter*, are phylogenetically distinct. *Carnimonas nigrificans* and *Zymobacter palmae* are the closest phylogenetic relatives of *H. alkalilenta*, and were isolated from cured meat and palm sap respectively [7, 8]. *H. alkalilenta* differs from *C. nigrificans* in its higher DNA G+C content and salt upper limit for growth, colony color, motility, its ability to grow at 5 °C and 37 °C, to utilize mannitol, in its inability to hydrolyze starch, to deaminize phenylalanine and to produce acids from D-mannitol and sucrose, in the proportion of the major membrane fatty acids and in the presence/absence of C_10:0_, C_12:0_, C_12:02_-OH, C_14:0_, C_16:0_ 3-OH, cyclo-C_17:0_, C_18:0_ and C_18:1_t9 [1, 8]. *H. alkalilenta* can be distinguished from *Z. palmae* in its higher DNA G+C content, colony color, pH and emperature range for growth, optimum growth temperature, its higher D-glucose tolerance, its ability to utilize citrate, its inability to give positive methyl red and Voges-Proskauer reactions, in the proportion of the major membrane fatty acids and in the presence/absence of C_10:0_, C_10:0_ 3-OH, C_12:0_ 2-OH, C_15:0_, C_17:0_, cyclo-C_17:0_, C_18:1_ ω9 and C_18: 1_ ω7 [1, 7].

Here, a summarized classification and key characteristics are presented for *H. alkalilenta* AW-7^T^, together with the description of the high-quality permanent draft genome sequence and annotation.

## Organism information

### Classification and features

The 16S rRNA gene sequence of *H. alkalilenta* AW-7^T^ was compared using NCBI BLAST under default settings (e.g., considering only the high-scoring segment pairs (HSPs) from the best 250 hits) with the most recent release of the Greengenes database [17] and the relative frequencies of taxa and keywords (reduced to their stem [18]) were determined and weighted by BLAST scores. The frequency of genera that belonged to the family *Halomonadaceae* was 95.2 %. The closest match of *H. alkalilenta* AW-7^T^ in 16S rRNA gene, submitted in INSDC (=EMBL/NCBI/DDBJ) under the accession number DQ421388 (=NR_043806), were *Zymobacter palmae*ATCC 51623^T^ (NR_041786) [7] and *Carnimonas**nigrifaciens* CTCBS1^T^ (NR_029342) [8] showing BLAST similarities of 96.2 % and 95.3 % respectively and HSP coverages of 99.7 % and 100 % respectively.

**Fig. 1:**
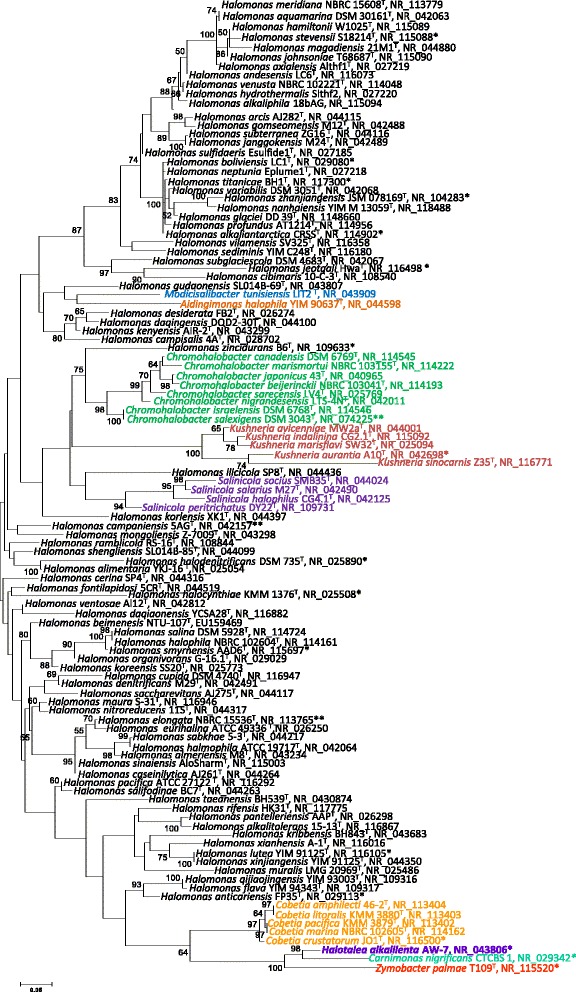
Phylogenetic tree displaying the position of *H. alkalilenta* AW-7^T^ among the type strains of other species within the *Halomonadaceae*. The tree was inferred from 1152 aligned characters [38, 39] of the 16S rRNA gene sequence under the maximum likelihood (ML) criterion [40]. Tree branches are constructed on the basis of the expected number of substitutions per site. Values above branches denote support values from 100 ML bootstrap replicates [41]. Members of different genera within the *Halomonadaceae* are depicted in different fonts color. Lineages with strain genome sequencing projects registered in GOLD [24] are labeled with one asterisk, and those also listed as ‘Complete and Published’ with two asterisks

Figure 1 shows the phylogenetic allocation of *H. alkalilenta* AW-7^T^ within the family *Halomonadaceae* in a 16S rRNA gene sequence-based tree. The sequence of the only 16S rRNA gene copy in the genome differs by 5 nucleotides from the previously published 16S rRNA sequence (DQ421388= NR_043806, coverage 95.0 %).

**Fig. 2:**
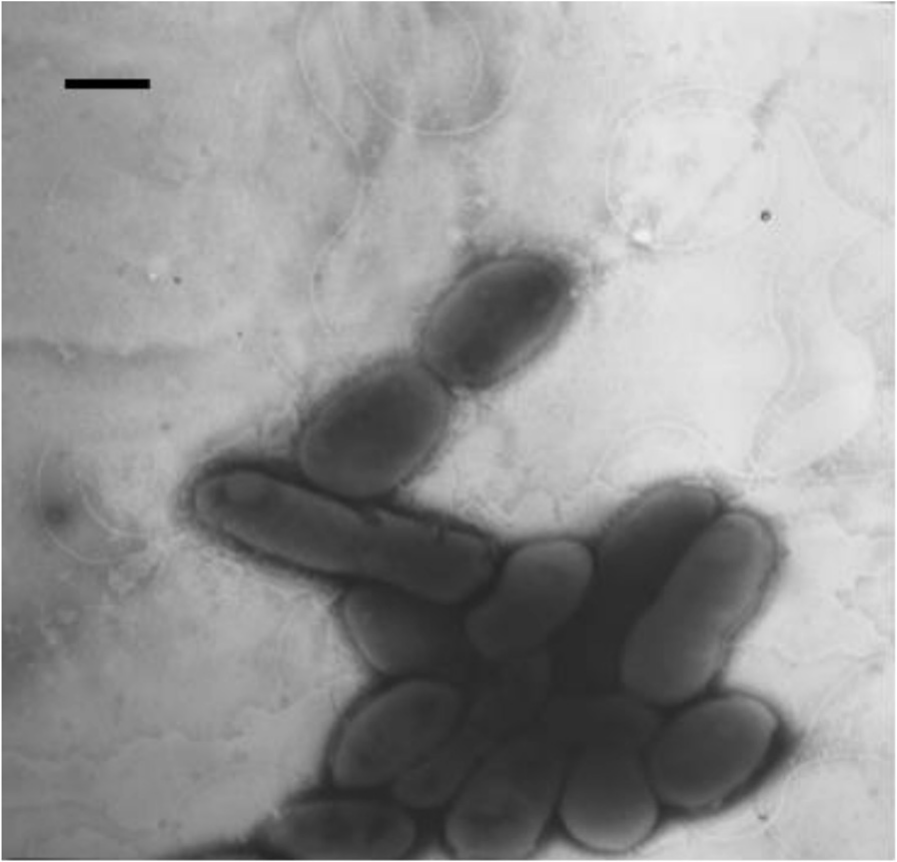
Electron micrograph of negatively-stained *H. alkalilenta* AW-7^T^ cells. Bar denotes 1 μm

*H. alkalilenta* AW-7^T^ is a Gram-negative motile rod-shaped bacterium [1] with a length of 1.4-2.1 μm and a width of 0.6-0.9 μm (Table 1 and Fig. 2). The temperature range for growth is 5–45 °C, with an optimum temperature for growth at 32–37 °C [1]. *H. alkalilenta* AW-7^T^ is halotolerant and alkalitolerant, growing at salinity and pH ranges of 0–150 g L^−1^ NaCl and 5–11, respectively [1]. The optimum salt and pH for growth are 0–3 % w/v NaCl and 7, respectively [1].

**Table 1:** Classification and general features of *Halotalea alkalilenta* strain AW-7^T^ according to the MIGS recommendations [42], published by the Genome Standards Consortium [43] and the Names for Life database [44]

MIGS ID	Property	Term	Evidence code^a^
	Classification	Domain *Bacteria*	TAS [45]
		Phylum *Proteobacteria*	TAS [46, 47]
		Class *Gammaproteobacteria*	TAS [47–49]
		Order *Oceanospirillales*	TAS [47, 50]
		Family *Halomonadaceae*	TAS [1–4, 51]
		Genus *Halotalea*	TAS [1]
		Species *Halotalea alkalilenta*	TAS [1]
		Type strain: *AW-7* ^*T*^	TAS [1]
	Gram stain	*negative*	TAS [1]
	Cell shape	*rod*	TAS [1]
	Motility	*motile*	TAS [1]
	Sporulation	*non-sporulating*	TAS [1]
	Temperature range	*5-45 °C*	TAS [1]
	Optimum temperature	*32-37 °C*	TAS [1]
	pH range; Optimum	*5-11; 7*	TAS [1]
	Carbon source	*carbohydrates, amino-acids, organic acid anions and alcohols*	TAS [1]
MIGS-6	Habitat	*olive mill waste*	TAS [1]
MIGS-6.3	Salinity	*up to 15 % NaCl w/v*	TAS [1]
MIGS-22	Oxygen requirement	*facultatively anaerobic*	IDA
MIGS-15	Biotic relationship	*free-living*	TAS [1]
MIGS-14	Pathogenicity	*none*	NAS
	Biosafety level	*1*	TAS [52]
MIGS-4	Geographic location	*Greece, Crete, Toplou Monastery*	TAS [1]
MIGS-5	Sample collection	*2003*	NAS
MIGS-4.1	Latitude	*35.220*	TAS [1]
MIGS-4.2	Longitude	*26.216*	TAS [1]
MIGS-4.3	Depth	*surface*	NAS
MIGS-4.4	Altitude	*161 m*	NAS

^a^Evidence codes - IDA: Inferred from Direct Assay; TAS: Traceable Author Statement (i.e., a direct report exists in the literature); NAS: Non-traceable Author Statement (i.e., not directly observed for the living, isolated sample, but based on a generally accepted property for the species, or anecdotal evidence). These evidence codes are from the Gene Ontology project [53]

*H. alkalilenta* AW-7^T^ is a non-denitrifying chemoorganotroph; it utilizes mostly L-glutamine and L-proline, followed by D-galactose, D-glucose, glycerol, D-mannitol, protocatechuate, L-serine, succinate and sucrose, while it grows weakly on acetate, citrate, D-fructose, maltose, sorbitol and gallate [1]. *H. alkalilenta* AW-7^T^ also produces acid aerobically from D-fructose, D-galactose, D-glucose, maltose, D-mannose and melibiose, and hydrolyses Tween 20 [1]. Despite the fact that urea hydrolysis is encoded in *H. alkalilenta* AW-7^T^ genome, no positive reaction was detected by Ntougias et al. [1] and the present study (using the EnteroPluri-Test). *H. alkalilenta* AW-7^T^ is susceptible to kanamycin, polymixin B, rifampicin, streptomycin and tetracycline (50 mg L^−1^ each) [1].

In the past, *H. alkalilenta* AW-7^T^ and *C. nigrificans* CTCBS1^T^ were reported as oxidase positive [1, 8]. However, genome comparisons showed that both *H. alkalilenta* AW-7^T^ and *C. nigrificans* CTCBS1^T^ possessed an identical oxidative phosphorylation pathway that lacks cytochrome c oxidase, which was distinct from that of *Z. palmae* T109^T^. In addition, no fermentation ability was previously detected for *H. alkalilenta* AW-7^T^ using standard incubation periods [1], although the pyruvate fermentation to acetate II MetaCyc pathway is encoded in both *H. alkalilenta* AW-7^T^ and *Z. palmae* T109^T^. For this reason, the fermentation ability of *H. alkalilenta* AW-7^T^ was re-examined under prolonged incubation period using the EnteroPluri-Test (BD, USA). No fermentation reaction was observed for incubations up to 4–days, although, thereafter, a positive reaction was obtained for glucose(at the 5th day of incubation, without gas production) and dulcitol (at 9th day of incubation). *H. alkalilenta* AW-7^T^ could not ferment adonitol, lactose, arabinose and sorbitol after a 9–days incubation period. In agreement to what was previously reported by Ntougias et al. [1], no growth of *H. alkalilenta* AW-7^T^ was observed in the present study on yeast extract-peptone-glucose agar plates placed for an incubation period of 1 month in an anaerobic jar containing the Anaerocult A system (Merck). However, exposure of culture plates to oxygen led to fastidious growth. In this sense, it is concluded that *H. alkalilenta* AW-7^T^ can tolerate anaerobic conditions through a slow fermentation mechanism.

#### Chemotaxonomy

The main membrane fatty acids of *H. alkalilenta* AW-7^T^ are in the descending order of concentration: C_18:1_ ω7c, C_16:0_, C_19:0_ cyclo ω8c, C_12:_0 3-OH and C_16:1_ ω7c/iso-C15:0 2-OH [1]. The only respiratory quinone found in *H. alkalilenta* AW-7^T^ is ubiquinone-9 [1].

## Genome sequencing and annotation

### Genome project history

*H. alkalilenta* AW-7^T^ was selected for sequencing on the basis of its phylogenetic position [19–21], and is part of Genomic Encyclopedia of Type Strains, Phase I: the one thousand microbial genomes project [22] which aims not only to increase the sequencing coverage of key reference microbial genomes [23]. The genome project is accessible in the Genomes On Line Database [24] and the entire genome sequence is deposited in GenBank. Sequencing, finishing and annotation were accomplished by the DOE Joint Genome Institute [25] using state of the art genome sequencing technology [26]. The project information is summarized in Table 2.

**Table 2:** *H. alkalilenta* AW-7^T^ genome sequencing project details

MIGS ID	Property	Term
MIGS-31	Finishing quality	High-Quality Draft
MIGS-29	Sequencing platforms	Illumina HiSeq 2000
MIGS-31.2	Sequencing coverage	300×
MIGS-30	Assemblers	vpAllpaths v. r46652
MIGS-32	Gene calling method	Prodigal 2.5
	INSDC ID	JHYY00000000
	Genbank Date of Release	May 5, 2014
	GOLD ID	Gp0040002
	NCBI project ID	221047
MIGS-13	Source material identifier	DSM 17697^T^
	Project relevance	GEBA-KMG, Tree of Life, Biodegradation, Extremophiles

### Growth conditions and genomic DNA preparation

*H. alkalilenta* AW-7^T^ was cultivated aerobically in trypticase soy yeast extract medium at 28 °C. Genomic DNA was obtained using the Invitrogen PureLink® Genomic DNA Mini Kit (Life Technologies Inc.) following the standard protocol. In addition, DNA prepared by the DSMZ is available via the DNA Bank Network [27].

### Genome sequencing and assembly

The draft genome of was generated at the DOE Joint Genome Institute using the Illumina technology [28]. An Illumina std shotgun library was constructed and sequenced using the Illumina HiSeq 2000 platform which generated 13,537,536 reads totaling 2,030.6 Mb. All general aspects of library construction and sequencing performed can be found at JGI website [29]. All raw Illumina sequence data was passed through DUK, a filtering program developed at JGI, which removes known Illumina sequencing and library preparation artifacts (Mingkun L, et al., unpublished). Following steps were then performed for assembly: (1) filtered Illumina reads were assembled using Velvet (version 1.2.07) [30], (2) 1–3 kb simulated paired end reads were created from Velvet contigs using wgsim [31], (3) Illumina reads were assembled with simulated read pairs using Allpaths–LG (version r46652) [32]. Parameters for assembly steps were: 1) Velvet (velveth: 63 –shortPaired and velvetg: −very clean yes –exportFiltered yes –min contig lgth 500 –scaffolding no –cov cutoff 10) 2) wgsim (−e 0 –1 100 –2 100 –r 0 –R 0 –X 0) 3) Allpaths–LG (PrepareAllpathsInputs:PHRED 64 = 1 PLOIDY = 1 FRAG COVERAGE = 125 JUMP COVERAGE = 25 LONG JUMP COV = 50, RunAllpathsLG: THREADS = 8 RUN = std shredpairs TARGETS = standard VAPI WARN ONLY = True OVERWRITE = True). The final draft assembly contained 56 contigs in 40 scaffolds, totaling 4.5 Kb in size. The final assembly was based on 1,500.0 Mb of Illumina data. Based on a presumed genome size of 5.0 Mb, the average input read coverage used for the assembly was 300.0 ×.

### Genome annotation

Genes were detected using the Prodigal software [33] at the DOE-JGI Genome Annotation pipeline [34, 35]. The CDSs predicted were translated and searched against the National Center for Biotechnology Information non-redundant database, UniProt, TIGRFam, Pfam, PRIAM, KEGG, COG, and InterPro databases. Additional gene prediction and functional annotation analysis was carried out in the Integrated Microbial Genomes – Expert Review platform [36]. The genome sequence and the annotations described in this paper are available from the Integrated Microbial Genome system [37].

## Genome properties

The genome is 4,467,826 bp long and comprised of 40 scaffolds with 64.62 % average GC content (Table 3). A total of 4,104 genes were predicted, consisting of 4,028 protein-coding and 76 RNA genes. The majority of protein-coding genes (87.79 %) were assigned to a putative function, whereas the remaining ones were annotated as hypothetical proteins. Distribution of genes into COGs functional categories is displayed in Table 4.

**Table 3:** Genome statistics

Attribute	Value	% of Total^a^
Genome size (bp)	4,467,826	100.00
DNA coding region (bp)	3,922,088	87.79
DNA G + C content (bp)	2,887,209	64.62
DNA scaffolds	40	
Total genes	4,104	100.00
RNA genes	76	1.85
tRNA genes	52	1.27
Protein-coding genes	4,028	98.15
Pseudo genes	0	0.00
Genes with function prediction (proteins)	3,603	87.79
Genes in paralog clusters	3,380	82.36
Genes assigned to COGs	3,246	79.09
Genes assigned Pfam domains	3,637	88.62
Genes with signal peptides	343	8.36
Genes with transmembrane helices	905	22.05
CRISPR repeats	3	

^a^The total is based on either the size of the genome in base pairs or the total number of protein coding genes in the annotated genome

**Table 4:** Number of genes associated with the general COG functional categories

Code	Value	% Age	Description
J	188	5.15	Translation, ribosomal structure and biogenesis
A	1	0.03	RNA processing and modification
K	313	8.58	Transcription
L	124	3.40	Replication, recombination and repair
B	3	0.08	Chromatin structure and dynamics
D	30	0.82	Cell cycle control, cell division, chromosome partitioning
V	29	0.79	Defense mechanisms
T	125	3.42	Signal transduction mechanisms
M	169	4.63	Cell wall/membrane biogenesis
N	58	1.59	Cell motility
U	56	1.53	Intracellular trafficking, secretion and vesicular transport
O	111	3.04	Posttranslational modification, protein turnover, chaperones
C	233	6.38	Energy production and conversion
G	259	7.10	Carbohydrate transport and metabolism
E	525	14.38	Amino acid transport and metabolism
F	86	2.36	Nucleotide transport and metabolism
H	164	4.49	Coenzyme transport and metabolism
I	120	3.29	Lipid transport and metabolism
P	261	7.15	Inorganic ion transport and metabolism
Q	98	2.68	Secondary metabolites biosynthesis, transport and catabolism
R	440	12.05	General function prediction only
S	257	7.04	Function unknown
-	858	20.91	Not in COGs

## Insights into the genome sequence

The genome size of *H. alkalilenta* AW-7^T^ (4.47 Mbp) is 50 % and 60 % greater than those of *Z. palmae* T109^T^ and *C. nigrificans* CTCBS1^T^ (2.73 and 2.98 Mbp) respectively. In *H. alkalilenta* AW-7^T^, protein coding genes involved in the major functional categories (i.e., amino acid, carbohydrate and lipid metabolism, membrane transport, energy metabolism) are 50 % and 30 % greater in number than those detected in *Z. palmae* T109^T^ and *C. nigrificans* CTCBS1^T^, respectively. Moreover, genes encoding xenobiotic metabolic proteins are 69 % and 57 % more in *H. alkalilenta* AW-7^T^ than those identified in *Z. palmae* T109^T^*and**C. nigrificans* CTCBS1^T^ respectively.

Genome data uncovered the genetic ability of *H. alkalilenta* AW-7^T^ to degrade several recalcitrant substrates. *H. alkalilenta* AW-7^T^ encodes the bioconversion of catechol and protocatechuate to β-ketoadipate via the β-ketoadipate and protocatechuate degradation II (ortho-cleavage) pathway respectively, as verified by the ability of strain AW-7^T^ to catabolize certain phenolic compounds. Aerobic benzoate degradation I is also encoded, permitting its catabolism via the catechol degrading pathway. Genes encoding fluoroacetate dehalogenase were identified in the genome of *H. alkalilenta* AW-7^T^, indicating its ability for fluoroacetate degradation. The detection of genes involved in cyanate and acrylonitrile degradation was also verified. Lastly, *H. alkalilenta* AW-7^T^ is genetically able to produce ectoine and glycine betaine, which appear to serve as the main osmolytes for the adaptation of this species under high osmotic conditions.

Based on genome metabolic features, *H. alkalilenta* AW-7^T^ is prototrophic for L-arginine, L-histidine, L-isoleucine, L-leucine, L-lysine, L-phenylalanine, L-tryptophan, L-tyrosine and L-valine auxotroph, and L-aspartate, L-glutamate, L-glutamine and glycine. Strain AW-7^T^ can synthesize selenocysteine but not biotin.

## Conclusions

Genome sequence and biochemical data of the highly osmotolerant species *Halotalea alkalilenta* AW-7^T^ revealed the presence of an oxidative phosphorylation pathway that lacks cytochrome c oxidase, and the encoding of the pyruvate fermentation to acetate II (MetaCyc pathway). *H. alkalilenta* AW-7^T^ could ferment glucose and ducitol after a prolonged incubation period, which is indicative of the induction of a slow fermentation mechanism, and results in the emendation of the genus *Halotalea* Ntougias et al. 2007. Comparisons to its closest phylogenetic relatives *Zymobacter palmae* T109^T^ and *Carnimonas nigrificans* CTCBS1^T^, confirm the distinct taxonomic position of *H. alkalilenta* AW-7 on the basis of its larger genome size and number of protein coding genes involved in the major functional categories and in xenobiotics metabolism. Furthermore, *H. alkalilenta* AW-7^T^ encodes the biotransformation of catechol and protocatechuate to β-ketoadipate via the β-ketoadipate and protocatechuate degradation II (ortho-cleavage) pathway respectively, verifying at the genome level the ability of strain AW-7^T^ to degrade phenolic compounds.

## Emended description of the genus *Halotalea* Ntougias et al. 2007

The description of the genus *Halotalea* is the one given by Ntougias et al. 2007 [1], with the following modification: Facultative anaerobe, which exhibits delayed glucose and dulcitol fermentation ability, and lacks cytochrome c oxidase activity.

## Abbreviations

KMG: One thousand microbial genomes
GEBA: Genomic encyclopedia of Bacteria and Archaea
MIGS: Minimum information about a genome sequence
TAS: Traceable
NAS: Non-traceable
